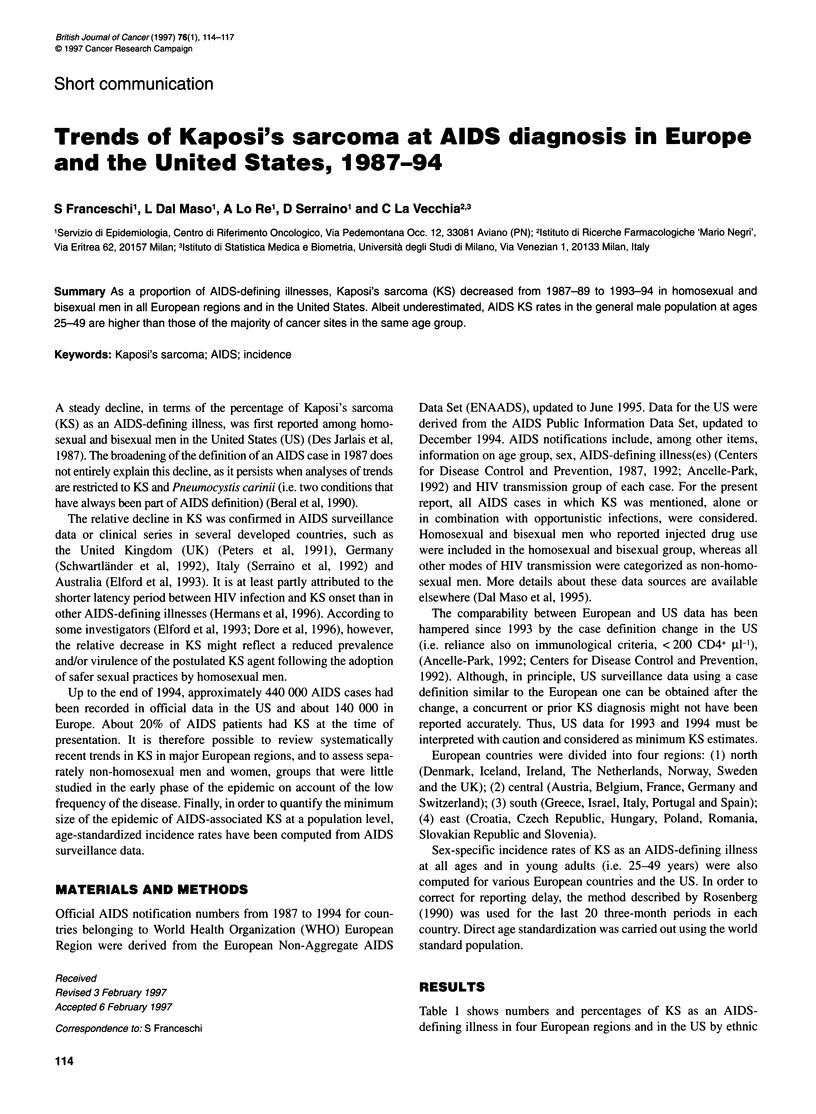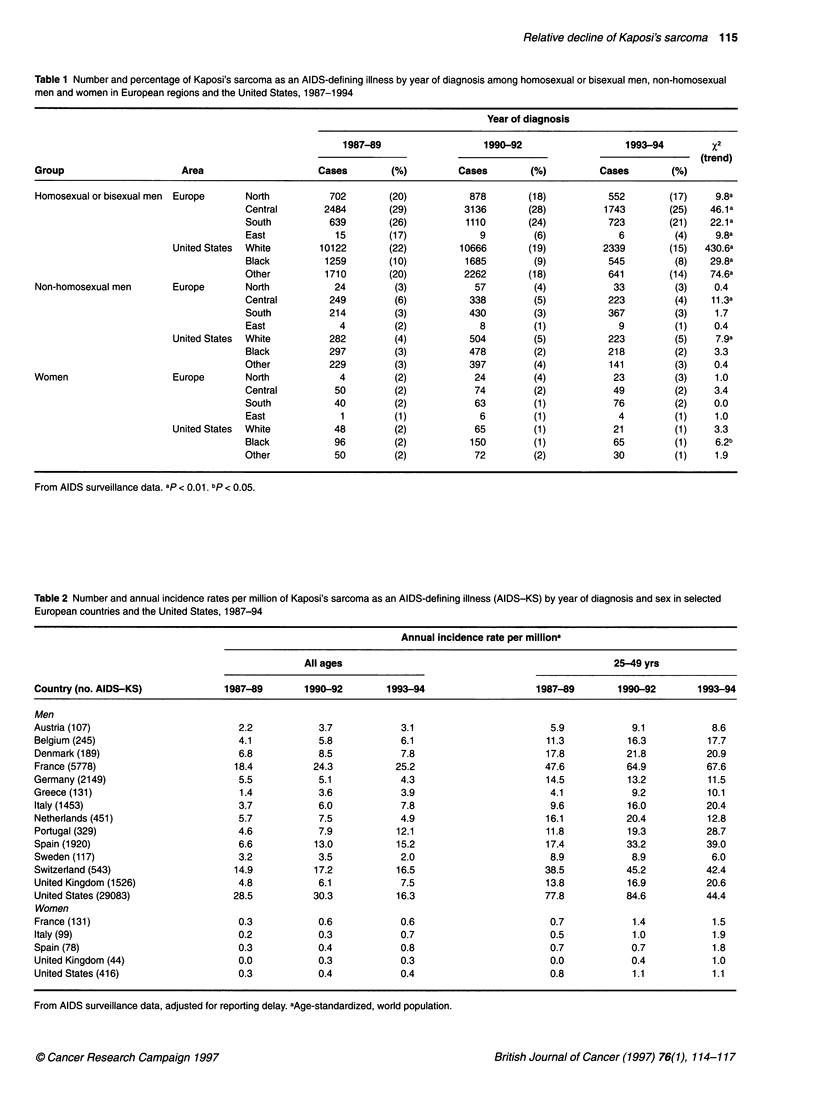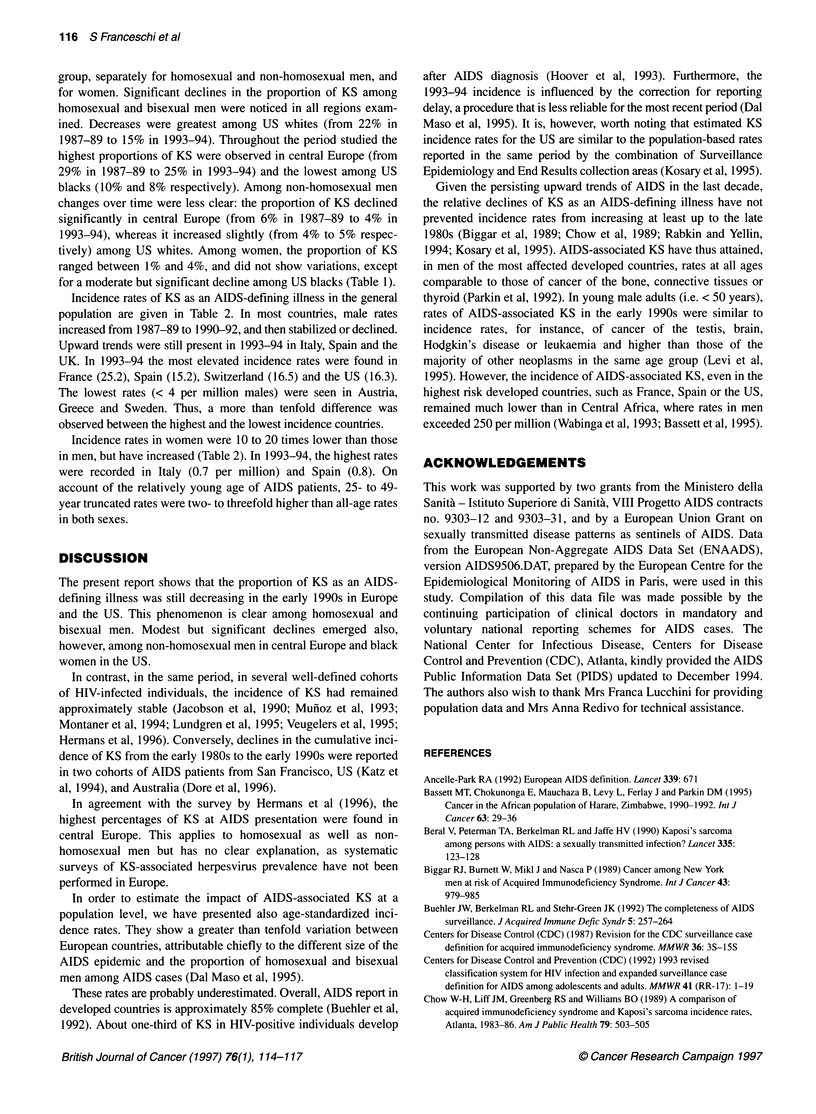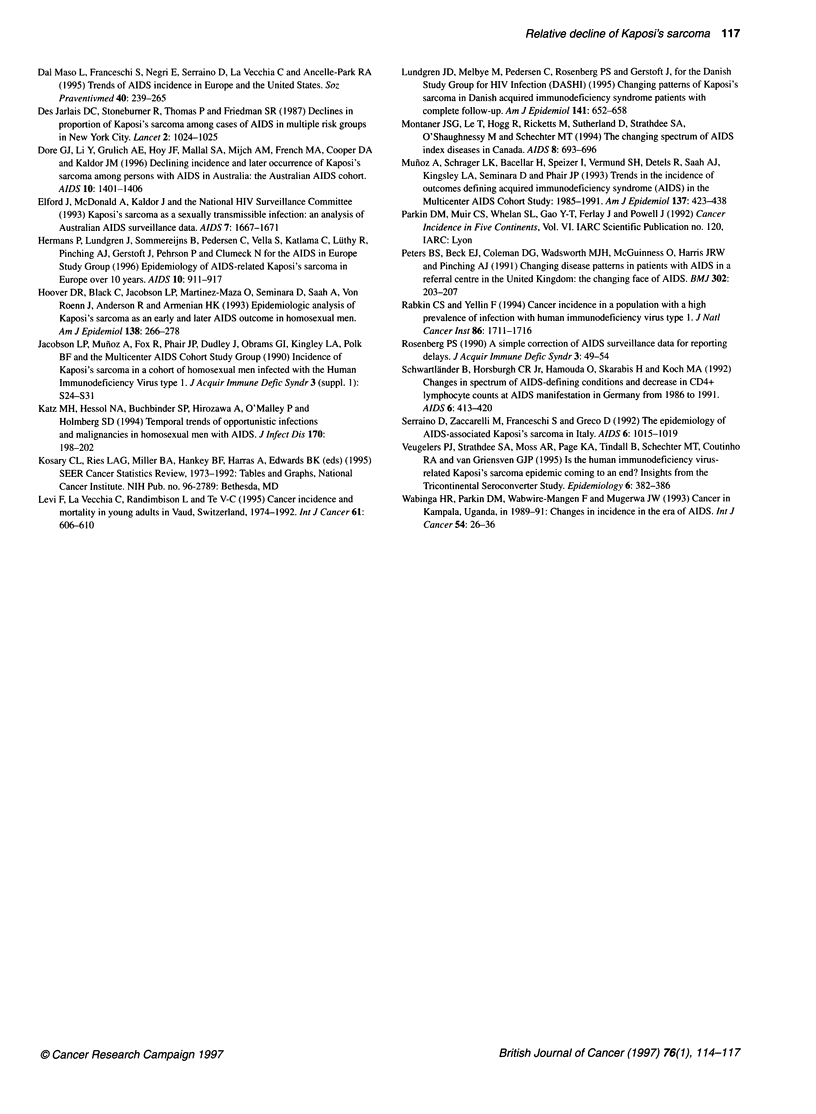# Trends of Kaposi's sarcoma at AIDS diagnosis in Europe and the United States, 1987-94.

**DOI:** 10.1038/bjc.1997.345

**Published:** 1997

**Authors:** S. Franceschi, L. Dal Maso, A. Lo Re, D. Serraino, C. La Vecchia

**Affiliations:** Servizio di Epidemiologia, Centro di Riferimento Oncologico, Aviano (PN), Italy.

## Abstract

As a proportion of AIDS-defining illnesses, Kaposi's sarcoma (KS) decreased from 1987-89 to 1993-94 in homosexual and bisexual men in all European regions and in the United States. Albeit underestimated, AIDS KS rates in the general male population at ages 25-49 are higher than those of the majority of cancer sites in the same age group.


British Journal of Cancer (1997) 76(1), 114–117
© 1997 Cancer Research Campaign

Short communication

### Trends of Kaposi's sarcoma at AIDS diagnosis in Europe and the United States, 1987–94

**S Franceschi[1], L Dal Maso[1], A Lo Re[1], D Serraino[1] and C La Vecchia[2,3]**

[1]Servizio di Epidemiologia, Centro di Riferimento Oncologico, Via Pedemontana Occ. 12, 33081 Aviano (PN); [2]Istituto di Ricerche Farmacologiche 'Mario Negri', Via Eritrea 62, 20157 Milan; [3]Istituto di Statistica Medica e Biometria, Università degli Studi di Milano, Via Venezian 1, 20133 Milan, Italy

**Summary** As a proportion of AIDS-defining illnesses, Kaposi's sarcoma (KS) decreased from 1987–89 to 1993–94 in homosexual and bisexual men in all European regions and in the United States. Albeit underestimated, AIDS KS rates in the general male population at ages 25–49 are higher than those of the majority of cancer sites in the same age group.

**Keywords:** Kaposi's sarcoma; AIDS; incidence

A steady decline, in terms of the percentage of Kaposi's sarcoma (KS) as an AIDS-defining illness, was first reported among homosexual and bisexual men in the United States (US) (Des Jarlais et al, 1987). The broadening of the definition of an AIDS case in 1987 does not entirely explain this decline, as it persists when analyses of trends are restricted to KS and *Pneumocystis carinii* (i.e. two conditions that have always been part of AIDS definition) (Beral et al, 1990).

The relative decline in KS was confirmed in AIDS surveillance data or clinical series in several developed countries, such as the United Kingdom (UK) (Peters et al, 1991), Germany (Schwartländer et al, 1992), Italy (Serraino et al, 1992) and Australia (Elford et al, 1993). It is at least partly attributed to the shorter latency period between HIV infection and KS onset than in other AIDS-defining illnesses (Hermans et al, 1996). According to some investigators (Elford et al, 1993; Dore et al, 1996), however, the relative decrease in KS might reflect a reduced prevalence and/or virulence of the postulated KS agent following the adoption of safer sexual practices by homosexual men.

Up to the end of 1994, approximately 440 000 AIDS cases had been recorded in official data in the US and about 140 000 in Europe. About 20% of AIDS patients had KS at the time of presentation. It is therefore possible to review systematically recent trends in KS in major European regions, and to assess separately non-homosexual men and women, groups that were little studied in the early phase of the epidemic on account of the low frequency of the disease. Finally, in order to quantify the minimum size of the epidemic of AIDS-associated KS at a population level, age-standardized incidence rates have been computed from AIDS surveillance data.

### MATERIALS AND METHODS

Official AIDS notification numbers from 1987 to 1994 for countries belonging to World Health Organization (WHO) European Region were derived from the European Non-Aggregate AIDS Data Set (ENAADS), updated to June 1995. Data for the US were derived from the AIDS Public Information Data Set, updated to December 1994. AIDS notifications include, among other items, information on age group, sex, AIDS-defining illness(es) (Centers for Disease Control and Prevention, 1987, 1992; Ancelle-Park, 1992) and HIV transmission group of each case. For the present report, all AIDS cases in which KS was mentioned, alone or in combination with opportunistic infections, were considered. Homosexual and bisexual men who reported injected drug use were included in the homosexual and bisexual group, whereas all other modes of HIV transmission were categorized as non-homosexual men. More details about these data sources are available elsewhere (Dal Maso et al, 1995).

The comparability between European and US data has been hampered since 1993 by the case definition change in the US (i.e. reliance also on immunological criteria, $< 200$ CD4$^+$ $\mu l^{-1}$), (Ancelle-Park, 1992; Centers for Disease Control and Prevention, 1992). Although, in principle, US surveillance data using a case definition similar to the European one can be obtained after the change, a concurrent or prior KS diagnosis might not have been reported accurately. Thus, US data for 1993 and 1994 must be interpreted with caution and considered as minimum KS estimates.

European countries were divided into four regions: (1) north (Denmark, Iceland, Ireland, The Netherlands, Norway, Sweden and the UK); (2) central (Austria, Belgium, France, Germany and Switzerland); (3) south (Greece, Israel, Italy, Portugal and Spain); (4) east (Croatia, Czech Republic, Hungary, Poland, Romania, Slovakian Republic and Slovenia).

Sex-specific incidence rates of KS as an AIDS-defining illness at all ages and in young adults (i.e. 25–49 years) were also computed for various European countries and the US. In order to correct for reporting delay, the method described by Rosenberg (1990) was used for the last 20 three-month periods in each country. Direct age standardization was carried out using the world standard population.

### RESULTS

Table 1 shows numbers and percentages of KS as an AIDS-defining illness in four European regions and in the US by ethnic

*Received*
*Revised 3 February 1997*
*Accepted 6 February 1997*

*Correspondence to:* S Franceschi

**Table 1** Number and percentage of Kaposi's sarcoma as an AIDS-defining illness by year of diagnosis among homosexual or bisexual men, non-homosexual men and women in European regions and the United States, 1987–1994

| | | | Year of diagnosis | | | | | | |
|---|---|---|---|---|---|---|---|---|---|
| | | | 1987–89 | | 1990–92 | | 1993–94 | | $\chi^2$ (trend) |
| Group | Area | | Cases | (%) | Cases | (%) | Cases | (%) | |
| Homosexual or bisexual men | Europe | North | 702 | (20) | 878 | (18) | 552 | (17) | 9.8[a] |
| | | Central | 2484 | (29) | 3136 | (28) | 1743 | (25) | 46.1[a] |
| | | South | 639 | (26) | 1110 | (24) | 723 | (21) | 22.1[a] |
| | | East | 15 | (17) | 9 | (6) | 6 | (4) | 9.8[a] |
| | United States | White | 10122 | (22) | 10666 | (19) | 2339 | (15) | 430.6[a] |
| | | Black | 1259 | (10) | 1685 | (9) | 545 | (8) | 29.8[a] |
| | | Other | 1710 | (20) | 2262 | (18) | 641 | (14) | 74.6[a] |
| Non-homosexual men | Europe | North | 24 | (3) | 57 | (4) | 33 | (3) | 0.4 |
| | | Central | 249 | (6) | 338 | (5) | 223 | (4) | 11.3[a] |
| | | South | 214 | (3) | 430 | (3) | 367 | (3) | 1.7 |
| | | East | 4 | (2) | 8 | (1) | 9 | (1) | 0.4 |
| | United States | White | 282 | (4) | 504 | (5) | 223 | (5) | 7.9[a] |
| | | Black | 297 | (3) | 478 | (2) | 218 | (2) | 3.3 |
| | | Other | 229 | (3) | 397 | (4) | 141 | (3) | 0.4 |
| Women | Europe | North | 4 | (2) | 24 | (4) | 23 | (3) | 1.0 |
| | | Central | 50 | (2) | 74 | (2) | 49 | (2) | 3.4 |
| | | South | 40 | (2) | 63 | (1) | 76 | (2) | 0.0 |
| | | East | 1 | (1) | 6 | (1) | 4 | (1) | 1.0 |
| | United States | White | 48 | (2) | 65 | (1) | 21 | (1) | 3.3 |
| | | Black | 96 | (2) | 150 | (1) | 65 | (1) | 6.2[b] |
| | | Other | 50 | (2) | 72 | (2) | 30 | (1) | 1.9 |

From AIDS surveillance data. [a]$P < 0.01$. [b]$P < 0.05$.

**Table 2** Number and annual incidence rates per million of Kaposi's sarcoma as an AIDS-defining illness (AIDS–KS) by year of diagnosis and sex in selected European countries and the United States, 1987–94

| | Annual incidence rate per million[a] | | | | | |
|---|---|---|---|---|---|---|
| | All ages | | | 25–49 yrs | | |
| Country (no. AIDS–KS) | 1987–89 | 1990–92 | 1993–94 | 1987–89 | 1990–92 | 1993–94 |
| *Men* | | | | | | |
| Austria (107) | 2.2 | 3.7 | 3.1 | 5.9 | 9.1 | 8.6 |
| Belgium (245) | 4.1 | 5.8 | 6.1 | 11.3 | 16.3 | 17.7 |
| Denmark (189) | 6.8 | 8.5 | 7.8 | 17.8 | 21.8 | 20.9 |
| France (5778) | 18.4 | 24.3 | 25.2 | 47.6 | 64.9 | 67.6 |
| Germany (2149) | 5.5 | 5.1 | 4.3 | 14.5 | 13.2 | 11.5 |
| Greece (131) | 1.4 | 3.6 | 3.9 | 4.1 | 9.2 | 10.1 |
| Italy (1453) | 3.7 | 6.0 | 7.8 | 9.6 | 16.0 | 20.4 |
| Netherlands (451) | 5.7 | 7.5 | 4.9 | 16.1 | 20.4 | 12.8 |
| Portugal (329) | 4.6 | 7.9 | 12.1 | 11.8 | 19.3 | 28.7 |
| Spain (1920) | 6.6 | 13.0 | 15.2 | 17.4 | 33.2 | 39.0 |
| Sweden (117) | 3.2 | 3.5 | 2.0 | 8.9 | 8.9 | 6.0 |
| Switzerland (543) | 14.9 | 17.2 | 16.5 | 38.5 | 45.2 | 42.4 |
| United Kingdom (1526) | 4.8 | 6.1 | 7.5 | 13.8 | 16.9 | 20.6 |
| United States (29083) | 28.5 | 30.3 | 16.3 | 77.8 | 84.6 | 44.4 |
| *Women* | | | | | | |
| France (131) | 0.3 | 0.6 | 0.6 | 0.7 | 1.4 | 1.5 |
| Italy (99) | 0.2 | 0.3 | 0.7 | 0.5 | 1.0 | 1.9 |
| Spain (78) | 0.3 | 0.4 | 0.8 | 0.7 | 0.7 | 1.8 |
| United Kingdom (44) | 0.0 | 0.3 | 0.3 | 0.0 | 0.4 | 1.0 |
| United States (416) | 0.3 | 0.4 | 0.4 | 0.8 | 1.1 | 1.1 |

From AIDS surveillance data, adjusted for reporting delay. [a]Age-standardized, world population.

© Cancer Research Campaign 1997

group, separately for homosexual and non-homosexual men, and for women. Significant declines in the proportion of KS among homosexual and bisexual men were noticed in all regions examined. Decreases were greatest among US whites (from 22% in 1987–89 to 15% in 1993–94). Throughout the period studied the highest proportions of KS were observed in central Europe (from 29% in 1987–89 to 25% in 1993–94) and the lowest among US blacks (10% and 8% respectively). Among non-homosexual men changes over time were less clear: the proportion of KS declined significantly in central Europe (from 6% in 1987–89 to 4% in 1993–94), whereas it increased slightly (from 4% to 5% respectively) among US whites. Among women, the proportion of KS ranged between 1% and 4%, and did not show variations, except for a moderate but significant decline among US blacks (Table 1).

Incidence rates of KS as an AIDS-defining illness in the general population are given in Table 2. In most countries, male rates increased from 1987–89 to 1990–92, and then stabilized or declined. Upward trends were still present in 1993–94 in Italy, Spain and the UK. In 1993–94 the most elevated incidence rates were found in France (25.2), Spain (15.2), Switzerland (16.5) and the US (16.3). The lowest rates (< 4 per million males) were seen in Austria, Greece and Sweden. Thus, a more than tenfold difference was observed between the highest and the lowest incidence countries.

Incidence rates in women were 10 to 20 times lower than those in men, but have increased (Table 2). In 1993–94, the highest rates were recorded in Italy (0.7 per million) and Spain (0.8). On account of the relatively young age of AIDS patients, 25- to 49-year truncated rates were two- to threefold higher than all-age rates in both sexes.

### DISCUSSION

The present report shows that the proportion of KS as an AIDS-defining illness was still decreasing in the early 1990s in Europe and the US. This phenomenon is clear among homosexual and bisexual men. Modest but significant declines emerged also, however, among non-homosexual men in central Europe and black women in the US.

In contrast, in the same period, in several well-defined cohorts of HIV-infected individuals, the incidence of KS had remained approximately stable (Jacobson et al, 1990; Muñoz et al, 1993; Montaner et al, 1994; Lundgren et al, 1995; Veugelers et al, 1995; Hermans et al, 1996). Conversely, declines in the cumulative incidence of KS from the early 1980s to the early 1990s were reported in two cohorts of AIDS patients from San Francisco, US (Katz et al, 1994), and Australia (Dore et al, 1996).

In agreement with the survey by Hermans et al (1996), the highest percentages of KS at AIDS presentation were found in central Europe. This applies to homosexual as well as non-homosexual men but has no clear explanation, as systematic surveys of KS-associated herpesvirus prevalence have not been performed in Europe.

In order to estimate the impact of AIDS-associated KS at a population level, we have presented also age-standardized incidence rates. They show a greater than tenfold variation between European countries, attributable chiefly to the different size of the AIDS epidemic and the proportion of homosexual and bisexual men among AIDS cases (Dal Maso et al, 1995).

These rates are probably underestimated. Overall, AIDS report in developed countries is approximately 85% complete (Buehler et al, 1992). About one-third of KS in HIV-positive individuals develop after AIDS diagnosis (Hoover et al, 1993). Furthermore, the 1993–94 incidence is influenced by the correction for reporting delay, a procedure that is less reliable for the most recent period (Dal Maso et al, 1995). It is, however, worth noting that estimated KS incidence rates for the US are similar to the population-based rates reported in the same period by the combination of Surveillance Epidemiology and End Results collection areas (Kosary et al, 1995).

Given the persisting upward trends of AIDS in the last decade, the relative declines of KS as an AIDS-defining illness have not prevented incidence rates from increasing at least up to the late 1980s (Biggar et al, 1989; Chow et al, 1989; Rabkin and Yellin, 1994; Kosary et al, 1995). AIDS-associated KS have thus attained, in men of the most affected developed countries, rates at all ages comparable to those of cancer of the bone, connective tissues or thyroid (Parkin et al, 1992). In young male adults (i.e. < 50 years), rates of AIDS-associated KS in the early 1990s were similar to incidence rates, for instance, of cancer of the testis, brain, Hodgkin's disease or leukaemia and higher than those of the majority of other neoplasms in the same age group (Levi et al, 1995). However, the incidence of AIDS-associated KS, even in the highest risk developed countries, such as France, Spain or the US, remained much lower than in Central Africa, where rates in men exceeded 250 per million (Wabinga et al, 1993; Bassett et al, 1995).

### ACKNOWLEDGEMENTS

This work was supported by two grants from the Ministero della Sanità – Istituto Superiore di Sanità, VIII Progetto AIDS contracts no. 9303–12 and 9303–31, and by a European Union Grant on sexually transmitted disease patterns as sentinels of AIDS. Data from the European Non-Aggregate AIDS Data Set (ENAADS), version AIDS9506.DAT, prepared by the European Centre for the Epidemiological Monitoring of AIDS in Paris, were used in this study. Compilation of this data file was made possible by the continuing participation of clinical doctors in mandatory and voluntary national reporting schemes for AIDS cases. The National Center for Infectious Disease, Centers for Disease Control and Prevention (CDC), Atlanta, kindly provided the AIDS Public Information Data Set (PIDS) updated to December 1994. The authors also wish to thank Mrs Franca Lucchini for providing population data and Mrs Anna Redivo for technical assistance.

### REFERENCES

Ancelle-Park RA (1992) European AIDS definition. *Lancet* **339**: 671

Bassett MT, Chokunonga E, Mauchaza B, Levy L, Ferlay J and Parkin DM (1995) Cancer in the African population of Harare, Zimbabwe, 1990–1992. *Int J Cancer* **63**: 29–36

Beral V, Peterman TA, Berkelman RL and Jaffe HV (1990) Kaposi's sarcoma among persons with AIDS: a sexually transmitted infection? *Lancet* **335**: 123–128

Biggar RJ, Burnett W, Mikl J and Nasca P (1989) Cancer among New York men at risk of Acquired Immunodeficiency Syndrome. *Int J Cancer* **43**: 979–985

Buehler JW, Berkelman RL and Stehr-Green JK (1992) The completeness of AIDS surveillance. *J Acquired Immune Defic Syndr* **5**: 257–264

Centers for Disease Control (CDC) (1987) Revision for the CDC surveillance case definition for acquired immunodeficiency syndrome. *MMWR* **36**: 3S–15S

Centers for Disease Control and Prevention (CDC) (1992) 1993 revised classification system for HIV infection and expanded surveillance case definition for AIDS among adolescents and adults. *MMWR* **41** (RR-17): 1–19

Chow W-H, Liff JM, Greenberg RS and Williams BO (1989) A comparison of acquired immunodeficiency syndrome and Kaposi's sarcoma incidence rates, Atlanta, 1983–86. *Am J Public Health* **79**: 503–505

© Cancer Research Campaign 1997

Dal Maso L, Franceschi S, Negri E, Serraino D, La Vecchia C and Ancelle-Park RA (1995) Trends of AIDS incidence in Europe and the United States. *Soz Praventivmed* **40**: 239–265

Des Jarlais DC, Stoneburner R, Thomas P and Friedman SR (1987) Declines in proportion of Kaposi's sarcoma among cases of AIDS in multiple risk groups in New York City. *Lancet* **2**: 1024–1025

Dore GJ, Li Y, Grulich AE, Hoy JF, Mallal SA, Mijch AM, French MA, Cooper DA and Kaldor JM (1996) Declining incidence and later occurrence of Kaposi's sarcoma among persons with AIDS in Australia: the Australian AIDS cohort. *AIDS* **10**: 1401–1406

Elford J, McDonald A, Kaldor J and the National HIV Surveillance Committee (1993) Kaposi's sarcoma as a sexually transmissible infection: an analysis of Australian AIDS surveillance data. *AIDS* **7**: 1667–1671

Hermans P, Lundgren J, Sommereijns B, Pedersen C, Vella S, Katlama C, Lüthy R, Pinching AJ, Gerstoft J, Pehrson P and Clumeck N for the AIDS in Europe Study Group (1996) Epidemiology of AIDS-related Kaposi's sarcoma in Europe over 10 years. *AIDS* **10**: 911–917

Hoover DR, Black C, Jacobson LP, Martinez-Maza O, Seminara D, Saah A, Von Roenn J, Anderson R and Armenian HK (1993) Epidemiologic analysis of Kaposi's sarcoma as an early and later AIDS outcome in homosexual men. *Am J Epidemiol* **138**: 266–278

Jacobson LP, Muñoz A, Fox R, Phair JP, Dudley J, Obrams GI, Kingley LA, Polk BF and the Multicenter AIDS Cohort Study Group (1990) Incidence of Kaposi's sarcoma in a cohort of homosexual men infected with the Human Immunodeficiency Virus type 1. *J Acquir Immune Defic Syndr* **3** (suppl. 1): S24–S31

Katz MH, Hessol NA, Buchbinder SP, Hirozawa A, O'Malley P and Holmberg SD (1994) Temporal trends of opportunistic infections and malignancies in homosexual men with AIDS. *J Infect Dis* **170**: 198–202

Kosary CL, Ries LAG, Miller BA, Hankey BF, Harras A, Edwards BK (eds) (1995) SEER Cancer Statistics Review, 1973–1992: Tables and Graphs, National Cancer Institute. NIH Pub. no. 96-2789: Bethesda, MD

Levi F, La Vecchia C, Randimbison L and Te V-C (1995) Cancer incidence and mortality in young adults in Vaud, Switzerland, 1974–1992. *Int J Cancer* **61**: 606–610

Lundgren JD, Melbye M, Pedersen C, Rosenberg PS and Gerstoft J, for the Danish Study Group for HIV Infection (DASHI) (1995) Changing patterns of Kaposi's sarcoma in Danish acquired immunodeficiency syndrome patients with complete follow-up. *Am J Epidemiol* **141**: 652–658

Montaner JSG, Le T, Hogg R, Ricketts M, Sutherland D, Strathdee SA, O'Shaughnessy M and Schechter MT (1994) The changing spectrum of AIDS index diseases in Canada. *AIDS* **8**: 693–696

Muñoz A, Schrager LK, Bacellar H, Speizer I, Vermund SH, Detels R, Saah AJ, Kingsley LA, Seminara D and Phair JP (1993) Trends in the incidence of outcomes defining acquired immunodeficiency syndrome (AIDS) in the Multicenter AIDS Cohort Study: 1985–1991. *Am J Epidemiol* **137**: 423–438

Parkin DM, Muir CS, Whelan SL, Gao Y-T, Ferlay J and Powell J (1992) *Cancer Incidence in Five Continents*, Vol. VI. IARC Scientific Publication no. 120, IARC: Lyon

Peters BS, Beck EJ, Coleman DG, Wadsworth MJH, McGuinness O, Harris JRW and Pinching AJ (1991) Changing disease patterns in patients with AIDS in a referral centre in the United Kingdom: the changing face of AIDS. *BMJ* **302**: 203–207

Rabkin CS and Yellin F (1994) Cancer incidence in a population with a high prevalence of infection with human immunodeficiency virus type 1. *J Natl Cancer Inst* **86**: 1711–1716

Rosenberg PS (1990) A simple correction of AIDS surveillance data for reporting delays. *J Acquir Immune Defic Syndr* **3**: 49–54

Schwartländer B, Horsburgh CR Jr, Hamouda O, Skarabis H and Koch MA (1992) Changes in spectrum of AIDS-defining conditions and decrease in CD4+ lymphocyte counts at AIDS manifestation in Germany from 1986 to 1991. *AIDS* **6**: 413–420

Serraino D, Zaccarelli M, Franceschi S and Greco D (1992) The epidemiology of AIDS-associated Kaposi's sarcoma in Italy. *AIDS* **6**: 1015–1019

Veugelers PJ, Strathdee SA, Moss AR, Page KA, Tindall B, Schechter MT, Coutinho RA and van Griensven GJP (1995) Is the human immunodeficiency virus-related Kaposi's sarcoma epidemic coming to an end? Insights from the Tricontinental Seroconverter Study. *Epidemiology* **6**: 382–386

Wabinga HR, Parkin DM, Wabwire-Mangen F and Mugerwa JW (1993) Cancer in Kampala, Uganda, in 1989–91: Changes in incidence in the era of AIDS. *Int J Cancer* **54**: 26–36

© Cancer Research Campaign 1997